# Domperidone inhibits cell proliferation via targeting MEK and CDK4 in esophageal squamous cell carcinoma

**DOI:** 10.1186/s12935-024-03291-8

**Published:** 2024-03-25

**Authors:** Qiang Yuan, Yunshu Shi, Yuhan Zhang, Yaqian Shi, Zubair Hussain, Jimin Zhao, Yanan jiang, Yan Qiao, Yaping Guo, Jing Lu, Ziming Dong, Zigang Dong, Junyong Wang, Kangdong Liu

**Affiliations:** 1 The Pathophysiology Department, School of Basic Medical Sciences, Zhengzhou University, Zhengzhou, 450000 Henan China; 2 China-US (Henan) Hormel Cancer Institute, Zhengzhou, 450000 Henan China; 3 State Key Laboratory of Esophageal Cancer Prevention and Treatment, Zhengzhou, 450000 Henan China; 4 Provincial Cooperative Innovation Center for Cancer Chemoprevention, Zhengzhou University, Zhengzhou, 450000 Henan China; 5 Cancer Chemoprevention International Collaboration Laboratory, Zhengzhou, 450000 Henan China

**Keywords:** MEK1/2, CDK4, Domperidone, ESCC

## Abstract

**Background:**

Esophageal squamous cell carcinoma (ESCC) is one of the leading causes of digestive system tumor related death in the world. Unfortunately, effective chemopreventive agent is lack for patients with ESCC in clinical practice, which leads to the extremely high mortality rate.

**Methods:**

A library of prescribed drugs was screened for finding critical anti-tumor properties in ESCC cells. The phosphoproteomics, kinase array, pulldown assay and drug affinity responsive target stabilization assay (DARTS) were applied to explore mechanisms and searched for synergistic targets. Established models of PDX in mice were used to determine the therapeutic effect of domperidone.

**Results:**

After screening a library of prescribed drugs, we discovered that domperidone has anti-tumor properties. Domperidone, acting as a gastroprokinetic agent, has been widely used in clinic for gastrointestinal motility disorders. Despite limited research, there are indications that domperidone may have anti-tumor properties. In this study, we determined that domperidone significantly inhibited ESCC proliferation in vitro and in vivo. We employed phosphoproteomics to reveal p-ERK, and p-SMAD3 down-regulation upon domperidone treatment. Then, the results of kinase assay and pulldown assay further validated that domperidone directly combined with MEK1/2 and CDK4, leading to the inhibition of their kinase activity. Furthermore, our results revealed that MEK/ERK and CDK4/SMAD3 signal pathway were major pathways in domperidone against ESCC.

**Conclusion:**

Collectively, these findings suggest that domperidone serves as an effective “multi-target” inhibitor of MEK1/2 and CDK4, offering potential benefits for the chemoprevention of ESCC.

**Supplementary Information:**

The online version contains supplementary material available at 10.1186/s12935-024-03291-8.

## Background

Esophageal cancer is a digestive system malignancy and ranks as the sixth leading cause of tumor-related mortality worldwide [1]. Esophageal Cancer is responsible for approximately 508,585 people deaths in 2018 worldwide [2, 3]. ESCC accounts for 90% of the total cases, which is recognized as primary subtype in Asia, particularly in China [4]. The currently available chemotherapies for ESCC, which are based on 5-fluorouracil or taxane plus platinum, offer limited survival benefits to patients [5]. In addition to these traditional therapies, various innovative therapeutic approaches have been also developed, including molecular-targeted agents and pembrolizumab monotherapy [6]. However, combination PD-1/PDL-1 inhibitor treatment for advanced ESCC has shown only a 9.9% objective response rate with a median overall survival of 5.8 months [7–10]. Therefore, finding novel and effective chemopreventive agents would be a rational approach to extend the survival of patients.

Since the approved drugs in clinic for cancer chemoprevention has been recognized as an effective and low-cost approach, for example, metformin and aspirin were repurposed for cancer prevention [11, 12]. Domperidone acts as a dopamine antagonist and has a special affinity with the D2 dopaminergic receptor (D2R) in the chemoreceptor trigger zone and gastrointestinal tract [13]. Domperidone increases antral-duodenal contractions and accelerates gastric emptying but has limited ability to penetrate the blood-brain barrier [14]. Hence, the agent has been used for amelioration of nausea, vomiting, and gastrointestinal motility disorders in clinical [15]. Although FDA has not acquired approval domperidone for treatment gastroparesis due to potential side effect of cardiac events for patients obtained with the medication intravenously, it also has been available in 58 countries as antiemetic [16, 17]. Moreover, in recent years, physicians have been permitted to prescribe domperidone to patients with gastrointestinal symptoms who have no response to standard treatment and are aged 12 years or older [16]. In addition, preclinical experiment showed that mice treated with domperidone have less liver tumorigenesis, suggesting its potential role as a chemopreventive agent [18]. Nevertheless, the effect of domperidone on ESCC and the potential mechanism of action have not been clearly elucidated.

MEK/ERK pathway has been identified as the most critical component of MAPK cascade [19]. MEK/ERK signaling cascade is hyperactivated in many cancer types, which controls tumor cells proliferation [20]. Multiple lines of evidence supported that targeting this pathway with MEK inhibitor shows therapeutic benefits for cancer [21, 22]. Trametinib, a MEK inhibitor, effectively suppresses the phosphorylation of ERK and subsequently inhibits downstream signaling molecules, thereby exerting an inhibitory effect on metastatic melanoma harboring BRAF(V600E/K) mutation [23]. Therefore, it is worth highlight that MEK could serve as a promising target for the chemoprevention of ESCC.

SMAD3 plays an essential role in medicating TGFβ growth antiproliferative effect, and meanwhile also acts as the only substrate for CDK4 [24]. When treated with TGFβ, SMAD2, SMAD3 with SMAD4 can form complex and accumulated in nuclear involving in transcriptional regulation of target genes, such as P21 and c-myc. Suppression c-myc transcription is necessary for SMAD3 antiproliferation response [25]. Upon c-myc downregulation, it can remove repression of P15 and P21 promoters, inducing P15 and P21 upregulation [24]. CDK4 phosphorylate SMAD3 at T8 and inhibits its transcriptional activity to promote the cell proliferation [26]. Especially, cancer cells possess highly levels CDK4. Therefore, evidence suggested that selecting CDK4 inhibitors through restoring SMAD3 antiproliferative function seems to be a promising strategy for patients.

In the current study, we investigated the inhibitory effect of domperidone on ESCC in vivo and in vitro. We also performed phosphoproteomics to explore the mechanism of domperidone against ESCC. Consequently, we verified p-ERK^T185/Y187^ and p-SMAD3^T8^ were down-regulated after domperidone treatment. Importantly, domperidone directly bound to MEK1/2 and depleted its kinase activity, thereby inducing anti-tumor effect via downregulation of MEK/ERK signal pathway. Meanwhile, the other target is CDK4, and domperidone can interact with CDK4. Domperidone blocked phosphorylation of SMAD3 by CDK4 to promote SMAD3 antiproliferation response, further inhibited the ESCC growth. “Double insurance” effect supports domperidone as a more selective approach for ESCC patients. Overall, our work identified domperidone as a promising multi-target chemopreventive agent for ESCC.

## Materials and methods

### Cell Culture

ESCC cell lines KYSE150 (RRID: CVCL_1348) and KYSE450 (RRID: CVCL_1353), and HEK293T (RRID: CVCL_0063) were obtained from the type culture collection of the Chinese Academy of Sciences (Shanghai, China). The Shantou human embryonic esophageal (SHEE) cell line was acquired from Dr. Enmin Li (Medical College of Shantou University) [27]. Cells also were authenticated by STR profiling and ensured to be free of mycoplasma. These cell lines have been authenticated in the past 3 years using short tandem repeat analysis. KYSE150 and KYSE450 cells were grown in RPMI-1640 medium with 10% FBS and all the cells were supplemented with 100 µg/mL streptomycin, 100 units/mL penicillin in a 37℃, 5% humidified environment. HEK293T cells were grown in DMEM with 10% FBS. Cells were utilized for experiments when they reached 70% confluence. All of cell lines were maintained within 10 passages.

### Regents and antibodies

Domperidone power was purchased from Med Chem Express (MCE), and was dissolved in DMSO. Domperidone was used at concentrations of 0, 10, 20, 40, and 60 µM in vitro. Domperidone for animal assay was purchased from Xi’an janssen pharmaceutical co. LTD. The dosages of domperidone for in vivo experiment were 25 mg/kg or 50 mg/kg. CNBr-Sepharose 4B beads were acquired from GE Healthcare. (E)-SIS3 was purchased from MCE.

### The antibodies

Phosphorylated ERK2(T185/Y187) was purchased from Thermo Fisher; phosphorylated SMAD3 (T8) and phosphorylated JUND (S100) were purchased from Abcam; The antibodies to detect P21, CyclinD1, CDK2, CDK4, c-myc and total ERK were acquired from Cell Signaling Technology; Total SMAD3 and total JUND were acquired from WanLeiBio. The active MEK1 and MEK2 human recombinant protein was purchased from Signal Chem.

### Cell proliferation assay

ESCC cells (KYSE150 and KYSE450) were seed into 96-well plates at a density of 1.0 × 10^3^ cells/well, and incubated for 16 h. And cells were subjected to varying concentrations of domperidone or a vehicle as control. After incubation for 24, 48, 72 and 96 h, the cells were washed with pre-cold PBS for 2 times. Then, cells were treated with 4% paraformaldehyde for 30 min at room temperature (RT). DAPI was applied to stain cell nucleus at 37℃ for 20 min and the cells were measured by IN Cell Analyze 6000.

### Anchorage-independent cell growth

A mixture comprising complete growth medium, including 10% FBS, 0.6% agar, and varying concentrations of domperidone, was prepared for the bottom layer in a 6-well plate. And cells (8 × 10^3^ cells/well) were routinely suspended in the top layer containing 10% FBS, 0.3% agar with various dose of domperidone. After incubation 10 days in incubator, the number of colonies was quantified and images were captured using the IN Cell Analyze 6000 system.

### Western blot

Cells were treated with or without 40 µM domperidone for 24 h, and then were lysed using RIPA buffer. Bichinconinic acid (BCA) assay kit was applied to measure the protein sample quantification, and each sample was prepared for same amount depending on protein concentration. Following SDS-PAGE separation, the proteins were subsequently transferred onto PVDF membranes (Millipore Corp). Subsequently, 5% non-fat milk was used to block the PVDF membranes for 1 h at RT, followed by incubation primary antibodies overnight at 4℃. Then, HRP-conjugated secondary antibodies were incubated for 2 h at RT. Specific bands were visualized by ECL luminescence reagent (Meilunbio).

### Sample preparation and phosphoproteome analysis

The detailed protocol was referenced as previously described [27]. KYSE150 cells were seeded in a 15 cm dish at a density of 4.0 × 10^6^ and exposure to 40 µM domperidone for 24 h, while the control group was treated with DMSO. The protein was obtained from cells using 8 M urea. After undergoing reduction using 5 mM dithiothreitol, the sample was exposed to 11 mM iodoacetamide for 15 min at RT in the absence of light. Peptides were separated by high-pH reverse-phase HPLC followed by trypsin digestion. Then, these peptides were analyzed by tandem mass spectrometry (MS/MS). Finally, the data were processed by Maxquant search engine (v.1.5.2.8).

### Pull down assay

Pull down assay was performed as previously described [28]. Either KYSE150 or KYSE450 cell lysates (1 mg) were incubated with domperidone-sepharose 4B beads in reaction buffer (50 mM Tris-HCl pH 7.5, 5 mM EDTA, 150 mM NaCl, 1 mM DTT, 0.01% NP-40 and 0.1 mM PMSF). The mixture underwent gentle shaking overnight at 4 °C, followed by three washes with wash buffer (50 mM Tris-HCl, pH 7.5, 5 mM EDTA, 150 mM NaCl, 1 mM DTT, 0.01% NP-40, and 0.2 mM PMSF). The bound proteins were eluted at 100 °C for 5 min using 6 × loading buffer. Western blot was employed to detect the bands.

### In vitro kinase assay

In vitro kinase assay was conducted according to the protocol of previous studies [29]. Briefly, the active MEK1, MEK2 (100 ng) was mixed with domperidone in kinase buffer (2 mM DTT), and kept incubation for 30 min at RT. Inactive ERK2 (500 ng) used as substrates and 200 µM ATP were added into the reaction buffer above and incubated for 30 min at 30℃. 10 µL 6 × Loading buffer was used to terminate the reaction, and the phosphorylation status of ERK was assessed using the p-ERK^T185/Y187^ antibody.

### Lentiviral infection and transfections

Both packaging vectors (pMD2.G, psPAX2) and virus vector (shMEK1, shMEK2, and sgCDK4) were transfected into HEK293T cells using the jetPRIME (PolyPlus). After transfection, the viral particles were collected after 24 h, 48 h and 72 h respectively, and filtered by a 0.45 µm filter. ESCC cells (KYSE150 and KYSE450) were seeded into 10 cm dish, and were infected with lentivirus medium in the presence of 8 µg/mL polybrene (Yeasen, China) for 24 h. Subsequently, cells were subjected to selection using 2 µg/mL puromycin (Solarbio) for a duration of 72 h, and the selected cells were utilized for subsequent experiments. The sequences were shown in“Supplementary Table 1”.

### Surface plasmon resonance (SPR)

SPR assay was performed as previously described [4]. Briefly, the Biacore T200 (GEHealthcare, England, UK) was used to perform SPR assays. MEK1 or MEK2 recombinant proteins were incubated on a CM5 sensor chip in PBS. Next, domperidone with different concentrations from 10 nM to 20 µM was injected. Then, the affinity of the agent and protein was measured in real time. Finally, the results were analyzed with the BIA evaluation 3.0 software.

### Drug affinity responsive target stabilization assay (DARTS)

KYSE 150 and KYSE450 cells were lysed with TTNE buffer (50 mM Tris-HCl pH 7.4, 0.15 mM NaCl; EDTA 2 mM) on ice, and the protein samples were quantified by BCA kit to 1 µg/µL. Subsequently, the drug was incubated with the protein for 30 min at RT. Protease was introduced at a 1:500 ratio to enzymatically digest the samples at RT. And then, 6 × Loading buffer was added, and the samples were subjected to boiling. Finally, Western blot was performed to check the ligand-protein target binding using corresponding antibody.

### Computer docking model

The crystal structures of MEK1 (PDB: 3EQF), MEK2 (PDB: 1S9I), and CDK4 (PDB: 2W99) from the PDB database were obtained. The structural formula for domperidone (Cas: 57808-66-9) was download from the PubChem Compound. Docking analysis was performed using the AutoDock 4.2.6. PyMOL was used to visualize ligand-protein complex.

### Wound healing assay

ESCC cells were incubated in 6-well plates at a density of 5 × 10^5^. When the cell confluence reaches 100%, cells were scratched using a pipette tip. The cells were incubated in RPMI-1640 medium without FBS, and subsequently cells were exposed to domperidone (20 µM or 40 µM). Photographs of the cells in the same area of the plates were captured after 12 and 24 h of incubation.

### Transwell migration assay

ESCC cells were incubated into 24-well plates with uncoated transwell chambers at a density of 2 × 10^4^. Cells were mixed with RPMI-1640 medium without FBS and the substratum was treated with RPMI-1640 medium containing 10% FBS and domperidone (20 µM or 40 µM) for 24 h whereas DMSO as control group. Then the cells were washed using PBS, and fixed with 4% paraformaldehyde following with 0.1% crystal violet staining. Finally, the transwell chambers was observed by microscope.

### Patient-derived esophageal xenograft model (PDX)

This project was acquired approval by the Ethics Committee of Zhengzhou University. The process of animal experiment was complied with ethical principle of medical research by national and international regulators. Severe combined immunodeficient mice (aged 6–8 weeks) were adopted in SPF condition. ESCC tissue from patients was carefully seeded into the lateral flanks of mice. When the average volume of tumor mass reached to 100 mm^3^, the mice were randomly divided into three groups for further experiment as following: untreated domperidone as vehicle group; 5 mg/kg domperidone group; 20 mg/kg domperidone group. The mice were given with domperidone or vehicle by gavage six times per week. The body weight of mice was recorded every week. Once the average volume of tumor mass reached to 1000 mm^3^, the experiment was terminated and the tumor tissues were extracted. The principle for calculating tumor volume was described: tumor volume (mm^3^) = (length × width × height × 0.5).

### Immunohistochemical (IHC) analysis

The tumor mass from PDX models were fixed and cut into 4 μm of thickness section. After heat-inducing epitope antigen retrieval was performed, 5% goat serums were administrated to block non-specific ionic bindings. The primary antibody was prepared and incubated overnight at 4℃, followed by secondary antibodies incubation for 30 min at 37℃. DAB reaction solution was added to the sections and monitored under microscope. Then, hematoxylin was used to visualize the staining of cell nucleus. The sectioned tissues were dehydrated through various grades of alcohol, cleared with xylene, and glass coverslips were put on the tissues. Images were analyzed by Image J pro.

### Statistical analysis

All results of this study were showed as the mean ± standard deviation (SD). SPSS 20.0 was used to assess statistical analysis. Student’s *t* test or one-way analysis of variance (ANOVA) was performed to compare significant differences. *P* < 0.05 was recognized as statistically significant, and * represented *P* < 0.05, ** represented *P* < 0.01 and *** represented *P* < 0.001. GraphPad Prism 8 was performed for graph creation.

## Results

### Domperidone suppresses proliferation of ESCC cells

**Fig. 1:**
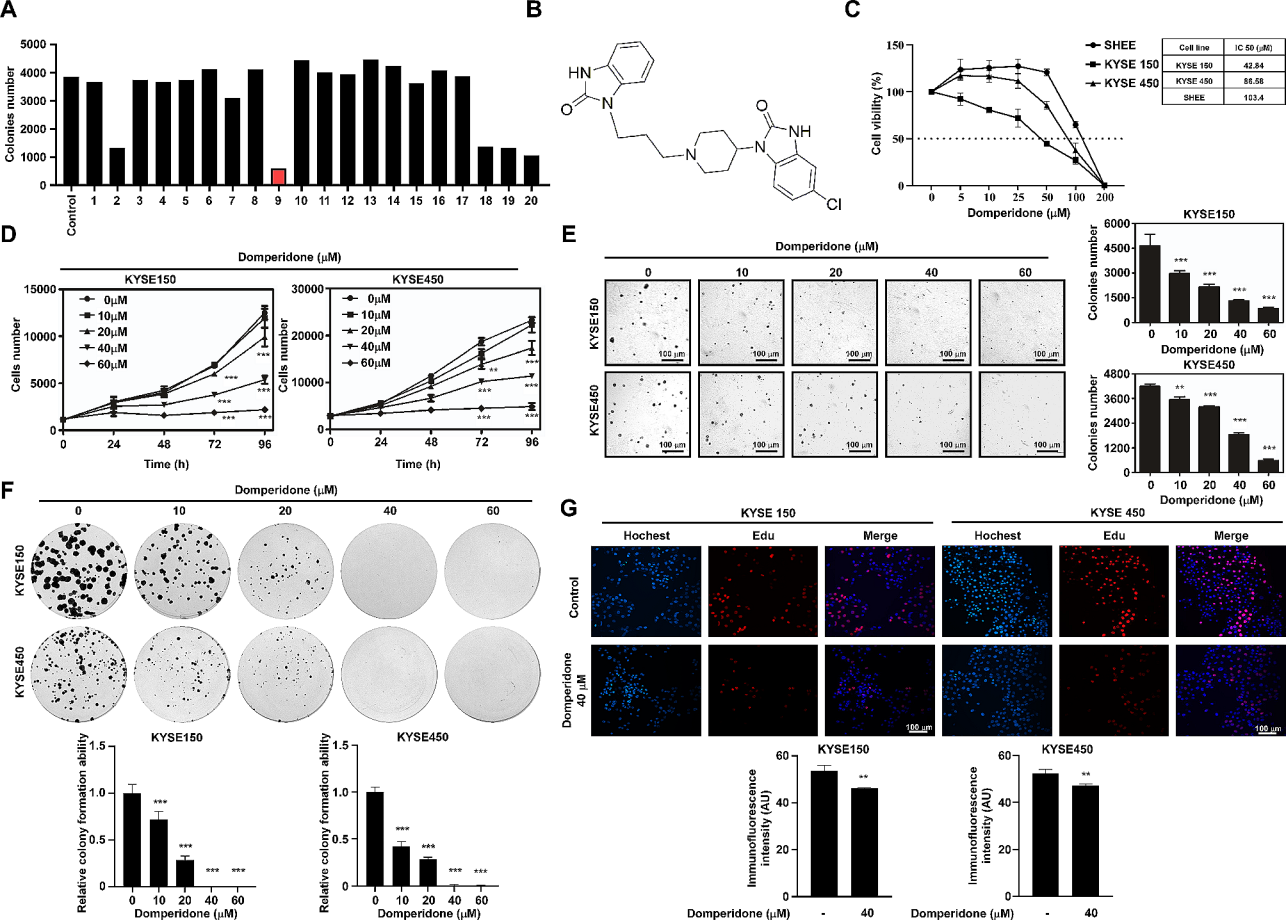
Domperidone effectively inhibits proliferation of ESCC cells (**A**) Domperidone was identified through soft agar assay from the clinical drug library. (**B**) The chemical structure of Domperidone is depicted. (**C**) KYSE150, KYSE450 and SHEE cells were treated with different doses of domperidone (0, 5, 10, 25, 50 100 and 200 µM) for 48 h, and IC 50 calculated by GraphPad Prism 8. (**D**) The human ESCC cell lines, KYSE150 (left panel) and KYSE450 (right panel), were exposed to various concentrations of domperidone or DMSO as control group for 24 h, 48 h, 72 and 96 h. The cell numbers were calculated and analyzed by IN Cell Analyzer 6000. (**E**) Anchorage-independent cell growth was assessed using a soft agar assay with different concentrations of domperidone (0, 10, 20, 40 and 60 µM). After incubation for 10 days, colonies were captured and analyzed using In Cell Analyze 6000. Results from three independent experiments were presented by comparing domperidone treatment group with control group. (**F**) Representative images obtained from the clone formation assay are shown. (**G**) KYSE150 (Left) and KYSE450 (right) were treated with 40 µM domperidone for 24 h, and then cell proliferation was detected by EdU staining. Data were showed as mean values ± SD. *P < 0.05, **P < 0.01, ***P < 0.001

In our study, we screened a panel of clinically available drugs and identified domperidone (compound 9) as a promising candidate with potent antitumor activity (Fig. 1A-B). Comparing with ESCC cell lines (KYSE150 and KYSE450), domperidone showed a highly IC50 value in esophageal epithelium cell line, SHEE (42.84 µM for KYSE150 and 86.58 µM for KYSE450, and 103.4 µM for SHEE) (Fig. 1C). To explore the impact of domperidone on growth of ESCC cells, ESCC cell lines were treated different concentrations of domperidone for cell proliferation assay. The results suggested that domperidone treatment significantly inhibited cell growth of KYSE150 and KYSE450 cells and showed a time- and concentration-response manner (Fig. 1D). In addition, we evaluated whether domperidone could dampen anchorage-independent growth of ESCC, and the results of soft agar assay indicated that domperidone significantly suppressed colony formation of KYSE150 (10 µM decreased 36.1% as well as 60 µM decreased 81.8%) and KYSE450 cells (10 µM decreased 15.3% as well as 60 µM decreased 86.1%) (Fig. 1E). Moreover, the results of colony formation assays also revealed that domperidone significantly inhibited clone numbers in ESCC cell lines, and there was almost no colony formation after 60 µM drug treatment (Fig. 1F). The results of EdU (5-ethynyl-2’-deoxyuridine) staining also verified domperidone obviously suppressed ESCC proliferation at 40 µM (Fig. 1G). Furthermore, domperidone suppressed the migration of ESCC cells (Supplementary Fig. 1A-B). Therefore, these results together indicated that domperidone showed a strong inhibitory effect on ESCC cells.

### Phosphoproteomics analysis of domperidone inducing molecular changes

**Fig. 2:**
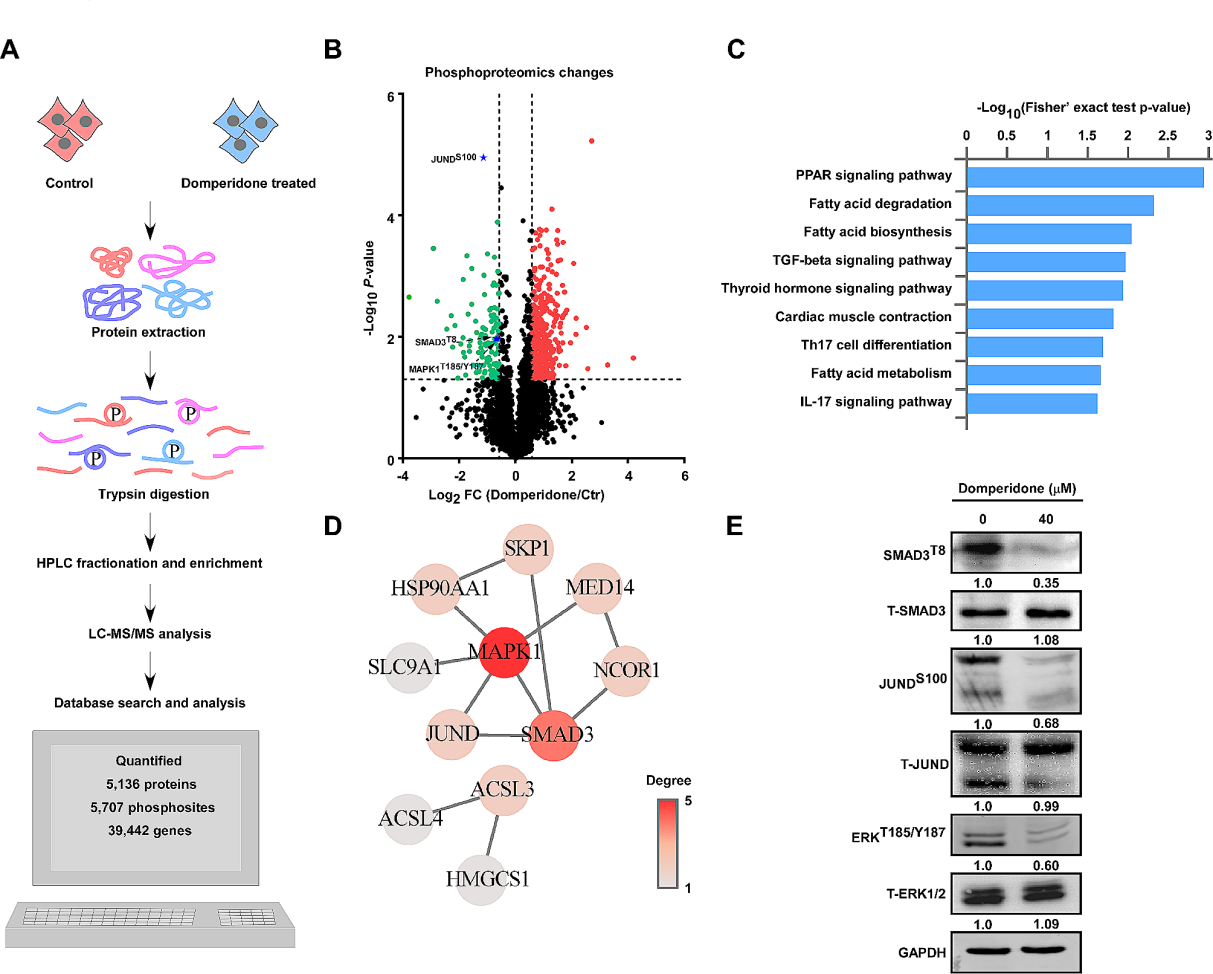
Phosphoproteomics analysis of domperidone inducing molecular changes. (**A**) Schematic representation of phosphorylated proteomics analysis in KYSE150 cells treated with 40 µM domperidone after 24 h. (**B**) Quantitative proteins were presented as a volcano plot, where down-regulated proteins are represented by green plots and up-regulated proteins by red plots. (**C**) KEGG pathway enrichment analysis was performed to identify down-regulated proteins in domperidone treatment group compared to control group. (**D**) A network illustrating major was presented, with circles representing down-regulated proteins after domperidone treatment. (**E**) Western blot results confirmed down regulation of phosphorylation sites, including p-SMAD3^T8^, p-ERK2^T185/Y187^ and p-JUND^S100^

To acquire a comprehensive molecular understanding of domperidone-induced inhibitory effect of growth, we compared phosphoproteomics profiles of control and domperidone-treated cells by mass spectrometry (Fig. 2A). This project successfully identified a total of·7152 phosphorylation sites covering more than 2500 phosphoproteins, of which 5707 phosphorylation sites were quantified. We strictly stuck to the quality filtering principles, fold change > 1.5 and *P*-value < 0.05, and a total of 454 phosphorylation sites were recognized significant differences between groups, including 336 sites up-regulated and 118 sites down-regulated (Fig. 2B). To acquire a complete overview of the function of molecular changes, KEGG pathway enrichment was performed and the result presented some enriched down-regulated pathways, including TGFβ signal pathway, Th17 cell differentiation, and IL17 signal pathway and so on (Fig. 2C). Major pathways involving the related annotated proteins were showed as a network, such as JUND, SMAD3, and MAPK1 (ERK2) (Fig. 2D). Phosphorylation sites of these proteins were confirmed by Western blot (Fig. 2E, Supplementary Fig. 5A). We confirmed that p-ERK^T185/Y187^, p-SMAD3^T8^ and p-JUND^S100^ were down-regulated and the results were consistent with phosphoproteomics analysis, which also verified the reliability of phosphoproteomics data.

### Domperidone directly binds to MEK1/2 and CDK4

The MEK acts as a direct upstream kinase of ERK2. We hypothesized whether domperidone could abolish the enhancement of ERK phosphorylation in MEK/ERK dependent manner by targeting MEK. Furthermore, phosphorylation of SMAD3 at Tyr8 (T8) was inhibited upon domperidone treatment, and we speculated whether this effect was due to the disturbance of upstream kinases activity. It has been indicated that only CDK4 could phosphorylate SMAD3 at T8 [24].

**Fig. 3:**
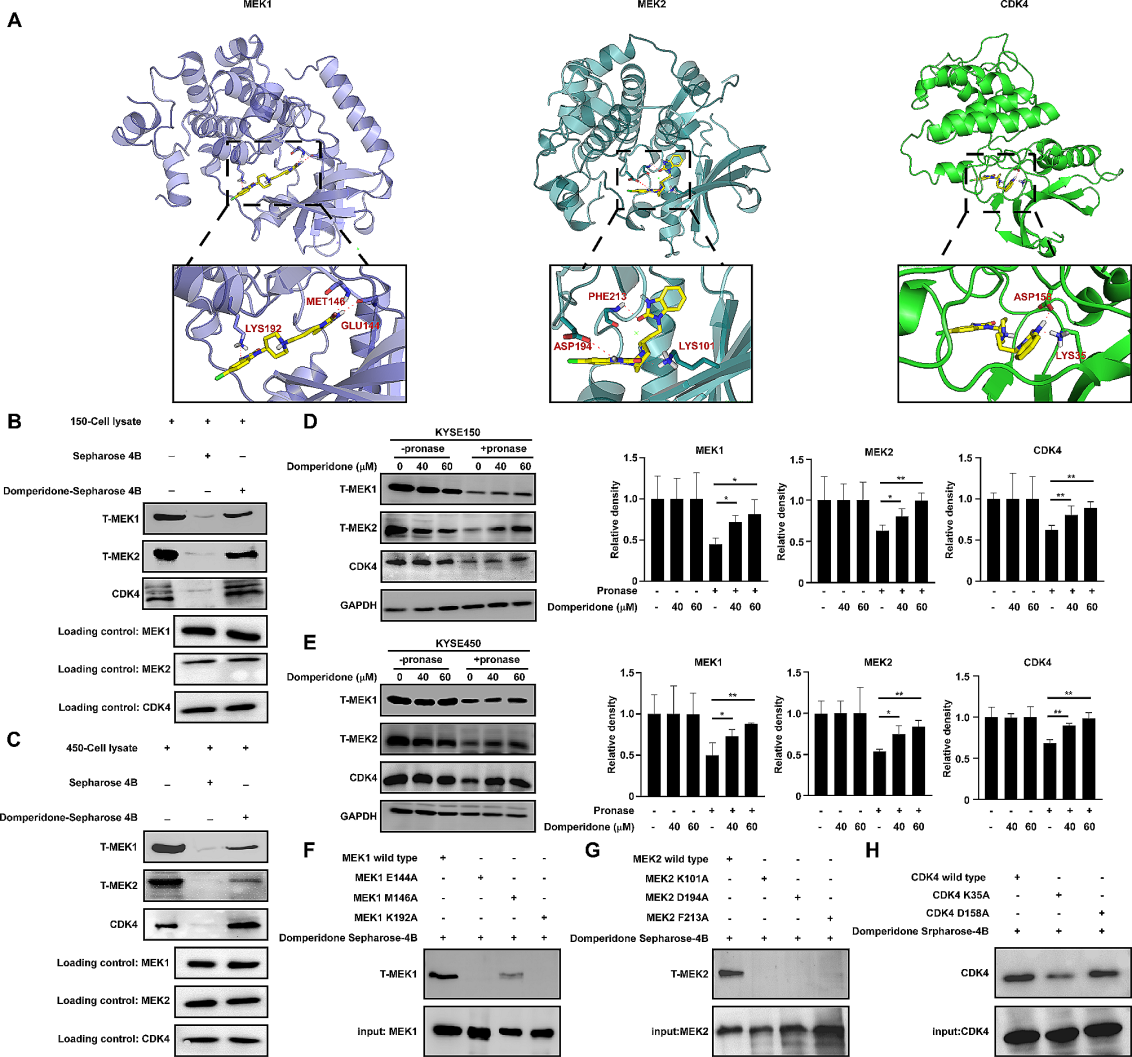
Domperidone directly binds to MEK1/2 and CDK4 (**A**) Docking model analysis illustrated the interaction between domperidone and MEK1/2 or CDK4. (**B**) KYSE150 or (**C**) KYSE450 cell lysate was incubated with domperidone-conjugated Sepharose 4B beads, MEK1/2 and CDK4 were pulled down through visualization using Western blot. Immunoblot analysis of pronase-digested KYSE150 (**D**) and KYSE450 (**E**) cell lysate showed the presence of MEK1/2 and CDK4. (**F**) Pull down assay was used to evaluate the binding affinity between domperidone and mutant MEK1. (**G**) Pull down assay was used to evaluate the binding affinity between domperidone and mutant MEK2. (**H**) Pull down assay was used to evaluate the binding affinity between domperidone and mutation CDK4.

To determine whether domperidone interacts with the MEK1/2 or CDK4, we conducted computational molecular docking assays. The results suggested that domperidone bound with MEK1/2 or CDK4 protein (Fig. 3A). To validate the affinity between domperidone and MEK1/2 or CDK4 predicted by the silico analyses, pull-down assay and DARTS assay were performed. The results suggested that domperidone could directly bind with MEK1, MEK2 and CDK4 in vitro (Fig. 3B-C). DARTS assay also validated that domperidone could directly interact with MEK1, MEK2 and CDK4 ex vivo (Fig. 33D-E). In addition, we used SPR assay to calculate the dissociation constant (KD value). The results revealed the KD value for interaction between domperidone and MEK1, MEK2 respectively, 6.2 µM and 6.66 µM (Supplementary Fig. 2A). In addition, a computer modeling approach was employed to establish the binding orientation between domperidone and MEK1, MEK2 and CDK4. The results showed that domperidone formed several contacts with MEK1, including LYS192, MET146 and GLU144 (Fig. 3A Left). Similarly, domperidone also established several contacts with MEK2, including LYS 101, PHE 213 and ASP194 (Fig. 3A middle). When we mutated these sites, we found that mutant version markedly reduced binding affinity with domperidone (Fig. 3F-G). Docking model also was performed to verify interaction between domperidone with CDK4, and calculate the exactly binding sites (Fig. 3A Right). CDK4 contains 2 potential binding sites, LYS35 and ASP158. When we transfected these mutation sites of CDK4, domperidone presented a more unfavorable affinity compared to WT, especially K35A (Fig. 3H).

### Domperidone inhibits MEK/ERK pathway and CDK4/SMAD3 pathway

**Fig. 4:**
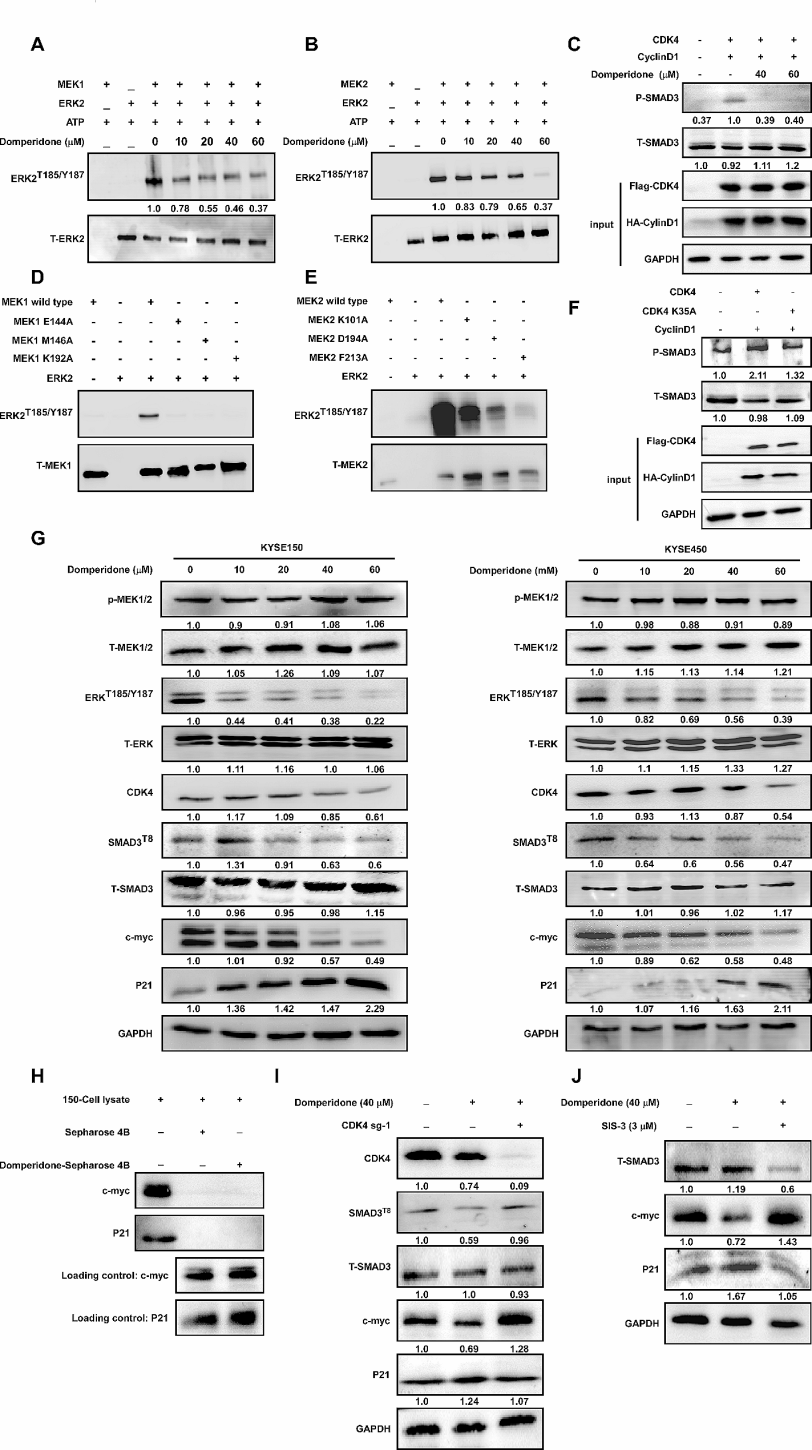
Domperidone inhibits MEK/ERK pathway and CDK4/SMAD3 pathway (**A**) MEK1 and MEK2 (**B**) kinase activity was assessed by an in vitro kinase assay using p-ERK^T185/Y187^ antibody. Active MEK1 or MEK2 incubated with inactive ERK2 in kinase reaction buffer together with different dose of domperidone and then followed by Western blot analysis. (**C**) KYSE150 cells were co-transfected with CDK4-Flag and CylinD1-HA, and then treated with 40 µM or 60 µM domperidone for 24 h. Cell lysates were immunoblotted with anti-p-Smad^T8^ antibody. Mutation version MEK1 (**D**) or MEK2 (**E**) incubated with inactive ERK2 in kinase reaction buffer, followed by Western blot analysis. (**F**) KYSE150 cells were co-transfected with either CDK4-Flag or CDK4 K35A-Flag along with CylinD1-HA. Cell lysates were immunoblotted with anti-p-SMAD^T8^ antibody. (**G**) KYSE150 and KYSE450 cells were treated with different concentration of domperidone after 24 h. Protein levels of P-ERK, p-MEK1/2, T-ERK, T-MEK1/2, p-SMAD3, T-SMAD3, CDK4, P21 and c-myc were analyzed by Western blot. (**H**) Pull down assay was used to evaluate the binding affinity between domperidone and c-myc or P21. (**I**) KYSE150 cells and CDK4 knock out cells were treated with 40 µM domperidone for 24 h, and then Western blot was performed. (**J**) KYSE150 was incubated with 40 µM domperidone or domperidone and SMAD3 inhibitor (E)-SIS3 3µM for 24 h simultaneously.

Due to MEK is well-known kinase for ERK, we utilized in vitro kinase assay to evaluate the inhibitory MEK activity by domperidone. The results showed that domperidone inhibited MEK1/2 activity in a dose-dependent manner (Fig. 4A-B, Supplementary Fig. 5B-C). Upon mutation these sites, we acquired the same effect with domperidone treatment (Fig. 4D-E). The Cyclin D1 can bind and activate CDK4, which in turn can phosphorylate SMAD3 at T8 [24]. Therefore, we transfected CDK4 and CylinD1 with domperidone treatment, and the results showed that domperidone inhibited phosphorylate SMAD3, which indicated CDK4 activity was down-regulated in a domperidone-dependent manner (Fig. 4C, Supplementary Fig. 5D). We further evaluated whether mutation K35 could disturb CDK4 carrying out kinase function. We found that the mutation K35 reduced the kinase activity of CDK4 with a reduced phosphorylation of SMAD3 (Fig. 4F, Supplementary Fig. 5E).

Further, we detected whether the MEK1/2 downstream signaling in ESCC cells could be obstructed by domperidone treatment. As predicted, phosphorylation of ERK^T185/Y187^ were progressively down-regulated in response to various dose of domperidone (Fig. 4G, Supplementary Fig. 5F). Immunofluorescence results also were consistent with results above (Supplementary Fig. 2B). Furthermore, we observed that domperidone efficiently abolished the phosphorylation of CDK4 on SMAD3 in a concertation-dependent manner (Fig. 4G, Supplementary Fig. 5F). Inhibiting the CDK4-mediated phosphorylation of SMAD3 at T8 can impede its transcriptional activity, resulting in decreased c-myc expression and increased P21 expression. Therefore, we detected CDK4/ SMAD3 pathway changes after domperidone treatment. We observed a dose-dependent decrease in SMAD3 phosphorylation levels upon administration of domperidone, accompanied by an increase in P21 expression and a decrease in c-myc expression compared to the untreated group (Fig. 4G). In addition, domperidone was unable to bind to the P21 and c-myc directly (Fig. 4H). Compared with just domperidone treatment group, c-myc, P21 and the phosphorylation of SMAD3 was rescued after domperidone incubation due to CDK4 knock down (Fig. 4I, Supplementary Fig. 5G). Furthermore, similar results were observed when administering SMAD3 inhibitor (E)-SIS3 and domperidone simultaneously (Fig. 4J, Supplementary Fig. 5H).

Collectively, these results demonstrate that domperidone directly suppresses MEK1/2 activity via interact with them. Meanwhile, the CDK4 inhibitory function of domperidone leads to down-regulation of p-Smad3, thereby disturbing its transcriptional activity.

### Reduction of cell proliferation by domperidone is dependent on MEK1/2 and CDK4

**Fig. 5:**
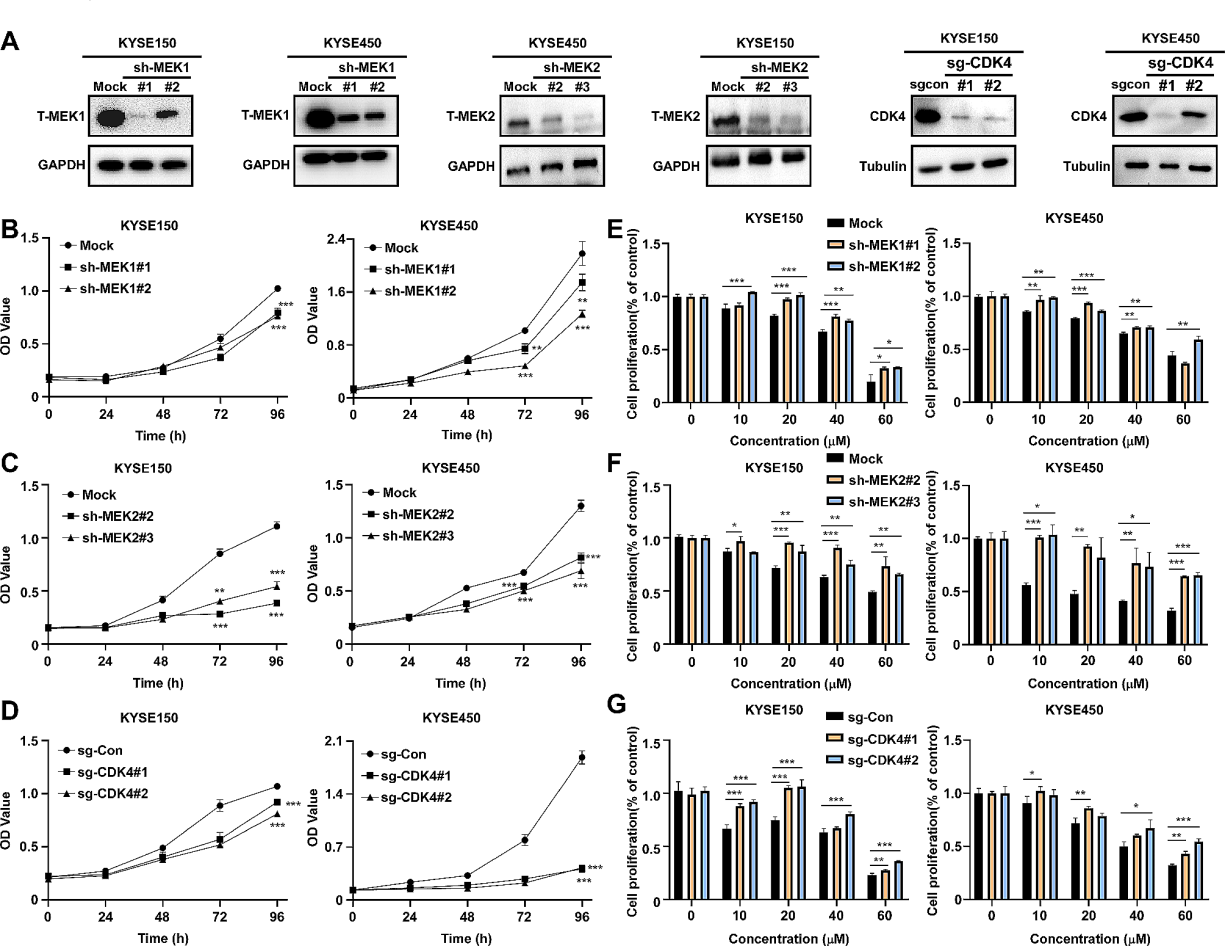
Reduction of cell proliferation by domperidone is dependent on MEK1/2 and CDK4 (**A**) Cell lines with knock down of MEK1, MEK2 or CDK4 were established, and the expression levels of MEK1, MEK2 or CDK4 were determined by Western blot. MTT assay was used to evaluate cell proliferation (**B**, **C**, **D**). The MEK1 (**E**), MEK2 (**F**) or CDK4 (**G**) knock down cell lines were treated with domperidone for 48 h, and the inhibitory rates were calculated. Data were showed as mean values ± SD. *P < 0.05, **P < 0.01, ***P < 0.001

To further assess the effect of MEK1/2 and CDK4 on the growth of ESCC, we respectively generated cell lines with MEK1, MEK2, or CDK4 knockdown (Fig. 5A). The results indicated when depriving MEK1, MEK2 or CDK4 expression, the proliferation of ESCC and colony formation ability were reduced (Fig. 5B-D, Supplementary Fig. 3). Since MEK1/2 and CDK4 serve as potential targets of domperidone, we employed cell lines with knockdown of either MEK1/2 or CDK4 to investigate whether the inhibitory growth effect induced by domperidone was mediated through modulation of MEK1/2 or CDK4 expression. The results showed that the suppressive effect of domperidone on tumor growth restored in cell lines with knockdown of MEK1, MEK2, or CDK4 (Fig. 5E-G). These data provided a molecular basis for considering domperidone as a “multi-target” anti-tumor agent via MEK1/2 and CDK4.

### Domperidone attenuates tumor growth of ESCC in PDX model

**Fig. 6:**
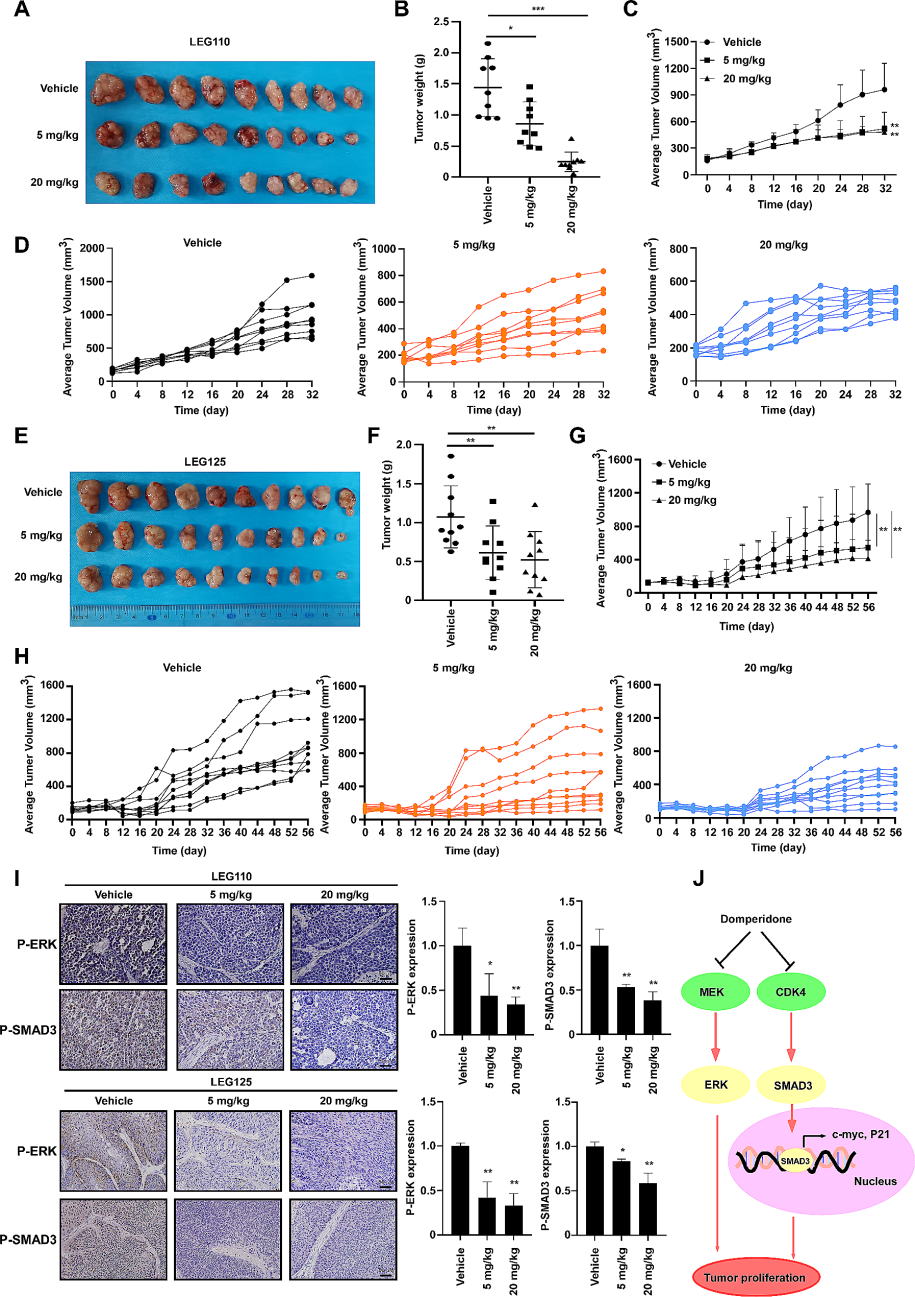
Domperidone suppresses ESCC tumor growth in PDX model The tumor tissues obtained from patients were subcutaneously injected into SCID mice, and then mice were given with domperidone (5 mg/kg, 20 mg/kg) or vehicle six times per week. (**A**, **E**) The mice were sacrificed and tumors mass were isolated from mice. (**C**, **D**, **G**, **H**) Tumor volume was monitored throughout the study period. Data are showed as mean ± S.D. **, *P* < 0.01 indicates a significant decrease between tumors from domperidone treatment and vehicle group. (**B**, **F**) Upon sacrifice of the mice, the weight of dissected tumor tissue was recorded. Data are showed as mean ± SD. *, *P* < 0.05, **, *P* < 0.01, ***, *P* < 0.001. (**I**) The expression of p-ERK and p-SMAD3 were examined in PDX model tumor tissue using IHC analysis (400×) (left panel). Quantification analysis of IHC staining results (Right panel). The positive rate was measured by Image J pro and represented as mean ± S.D. *, *P* < 0.05, **, *P* < 0.01. (**J**) Graphical conclusion for the findings of this work: domperidone bindings with MEK1/2 and CDK4, which disturbs their kinase activity in turn leads to suppress MEK/ERK and CDK4/SMAD3 pathway, and consequently inhibits ESCC proliferation.

To evaluate the in vivo anti-tumor effect of domperidone, we utilized a PDX model, which is commonly performed to predict personalized medicine strategies and evaluate preclinical drug activity [30]. Tumor tissues derived from patients with ESCC were orthotopically implanted into the back of mice. The mice received oral gavage administration of domperidone (5 mg/kg or 20 mg/kg) or vehicle six times per week. The results showed that domperidone obviously reduced tumor volumes and weights compared to vehicle group in two distinct cases, namely LEG110 and LEG125. For LEG110, average volume was 960.95 mm^3^ in vehicle group, 519.62 mm^3^ in 5 mg/kg group and 482.37 mm^3^ in 20 mg/kg group and domperidone exhibited a significant decrease in average tumor weights, and 5 mg/kg decreased 40.5% and 20 mg/kg decreased 83% (Fig. 6A-D). For LEG125, the average volume was 966.16 mm^3^ in vehicle group, 545.57 mm^3^ in 5 mg/kg group and 414.29 mm^3^ in 20 mg/kg group meanwhile 5 mg/kg decreased 40.5% and 20 mg/kg decreased 83% for tumor weights (Fig. 6E-H). Notably, there was no significant change in body weight among the groups (Supplementary Fig. 4A-B). Based on our preliminary results, domperidone exhibited obvious repressive effect against ESCC through modulation of the MEK/ERK and CDK4/SMAD3 axis in vitro. To validate these findings in vivo, tumor samples from mice were subjected to IHC analysis to detect p-ERK and p-SMAD3. These results showed that a significant reduction in the expression levels of p-ERK and p-SMAD3 following treatment with domperidone (Fig. 6I). These findings suggest domperidone reduces the growth promotional effect mediated by MEK and CDK4, thereby representing a promising chemoprevention agent for ESCC.

## Discussion

ESCC is a highly prevalent and lethal disease worldwide [31, 32]. Because of characteristic occult of the disease, patients with ESCC are usually found to be in the late-stage, which contributes to unsatisfied therapy effect and poor prognosis [33]. Despite recent advancements in ESCC diagnosis and treatment methods, the 5-year survival rate remains significantly low, with no approved chemoprevention agents in clinical settings [1, 34, 35]. Therefore, it is imperative to explore a novel agent for ESCC chemoprevention.

In this study, we have discovered that domperidone played a crucial role in inhibiting the growth of ESCC cells. Our results demonstrated that domperidone effectively inhibited ESCC cells growth and anchorage-independent growth in a time- and dose- dependent manner (Fig. 1D-G). Simultaneously, domperidone can markedly inhibit the migration of ESCC cells (Supplementary Fig. 1A-B). Next, the results revealed that even 5 mg/kg administration of domperidone significantly suppressed tumor growth compared with the vehicle-treated group in two individual PDX models (Fig. 6A-H), without causing noticeable body weight loss in mice. These findings indicate that domperidone can inhibit the proliferation of ESCC in vitro and in vivo, which holds the great potential for application in cancer chemoprevention. Recently, limited studies have also obtained attention about anti-tumor effect of domperidone. Domperidone induced triple-negative breast cancer cell apoptosis via JAK/STAT3 Signaling [35], and domperidone prevented males from hepatocellular carcinoma in a preclinical trial [36]. All these evidence supports that domperidone is an anti-tumor agent.

Subsequently, we compared the phosphoproteomics profiling analysis to explore growth-related molecular changes upon domperidone, and we focused on down-regulated pathways involving p-ERK^T185/187^ and p-SMAD^T8^ after domperidone treatment and these findings were validated using Western blot (Fig. 2D-E). According to the phosphoproteomics results above, we hypothesized that domperidone may attenuate the activity of their upstream kinase. MEK is known the most common kinase responsible for activating ERK signaling pathway while CDK4 exclusively phosphorylates SMAD3 T8 residue [29, 36]. Then, we employed a binding assay to confirm that the interaction of domperidone with upstream kinase, including MEK1/2 and CDK4 (Fig. 3) and transfection the mutant version of binding sites not only reduced affinity (Fig. 3F-H), but also suppressed MEK1/2 and CDK4 kinase activity, respectively (Fig. 4D-F). A growing body of evidence suggests that the development of single chemotherapeutics targeting multiple proteins, known as multi-target drugs, is recognized as a promising therapeutic strategy for cancer [37, 38]. Hence, we confirmed that domperidone, acting as a “multi-target” agent, bind with the active site of the protein (MEK1/2 and CDK4), thereby interfered with its kinase activity to exert an antiproliferation role in ESCC.

MEK/ERK pathway is closely associated with tumor growth in multiple cancer types, including ESCC [39]. Several MEK inhibitors have been approved in clinical use such as trametinib. However, resistance and toxicity issues limited their application [40]. Smad3 is acknowledged for its role in eliciting an antiproliferative response to TGFβ. Conversely, CDK4-mediated phosphorylation of SMAD3 at T8 promotes cell growth by suppressing its transcriptional activity, leading to upregulation of c-myc and downregulation of CDK inhibitor transcription (P21 and P15) [41]. Selective inhibition of CDK4 has also demonstrated efficacy in tumor treatment. Additionally, targeting CDK4 activity to modulate tumor-suppression SMAD3 may represent a promising strategy for cancer patients [36]. In recent years, combing with MEK and CDK4 inhibitor has more inhibitory proliferation effect for pancreatic ductal adenocarcinoma and RAS mutant colorectal cancer [42, 43]. Currently, combined MEKi (binimetinib) and CDK4/6i (palbociclib) obtained therapeutic efficacy with limitations of tolerability [43]. A novel dual kinase drug is need urgently. In this study, domperidone, acting as a dual kinase inhibitor, reduced MEK1/2 and CDK4 activity, leading to downregulation of p-ERK^T185/187^ or p-SMAD3^T8^ (Fig. 4A-C). Incubation of ESCC cell lines with domperidone resulted in dose-dependent downregulation of p-ERK, p-SMAD3, c-myc, along with upregulation of P21 expression (Fig. 4G). And it has been proved that domperidone directly disturbs MEK and CDK4 kinase activity to intercept downstream signaling pathways. Moreover, IHC analysis conducted on tumor tissues from PDX models also demonstrated a decrease in p-ERK, and p-SMAD3 levels following treatment with domperidone (Fig. 6I). There results indicated that domperidone inhibited tumor growth by suppressing MEK/ERK and CDK4/SMAD3 pathways via targeting MEK1/2 and CDK4.

While domperidone is already employed clinically as an agent for alleviating nausea and vomiting, concerns about its side effects have arisen. A meta-analysis revealed that patients receiving domperidone < 30 mg/d have been no observed QT interval prolongation and sudden cardiac death [44]. From a population-based nested case-control study, the risk of sudden cardiac death with domperidone administration is 1.71 versus non-use of study medications [45]. Furthermore, doctors are suggested to screen the patient using electrocardiograms to reduce the risk of sudden cardiac death. Recently, some professionals have suggested a careful assessment of risk factors in relation to the amelioration of gastrointestinal motility disorders in patients: (1) underlying cardiac diseases, especially QT prolongation, (2) co-administration QT-prolonging agents or CYP3A4 inhibitors [46]. (3) dosage < 30 mg/d in adult. Therefore, the side effects domperidone could be avoided through such a strict patient selection.

## Conclusion

In summary, this study has unveiled the anti-tumor effects of domperidone in ESCC. The antiproliferative activity of domperidone is achieved through its targeting of MEK1/2 and CDK4, resulting in the inhibition of the MEK/ERK and CDK4/SMAD3 pathways (Fig. 6J). Our findings suggest that domperidone holds promise as a “multi-target” chemopreventive agent for selected ESCC patients.

### Electronic supplementary material

Below is the link to the electronic supplementary material.

Supplementary Material 1

Supplementary Material 2

Supplementary Material 3

Supplementary Material 4

Supplementary Material 5

Supplementary Material 6

## Data Availability

No datasets were generated or analysed during the current study.
