# Supplementary material for: Domperidone inhibits cell proliferation via targeting MEK and CDK4 in esophageal squamous cell carcinoma
Source: Cancer Cell Int. 2024 Mar 25;24:114. doi: 10.1186/s12935-024-03291-8 (PMC10964662; doi:10.1186/s12935-024-03291-8)
Supplement: Supplementary file 1 — Supplementary Material 1 [file 12935_2024_3291_MOESM1_ESM.docx]

The primer sequences

Table 1. Sequences of oligonucleotides.

| Region | 5’-3’ Sequence |
| --- | --- |
| Sh-MEK1#1 F | CCGGGTCCTACATGTCGCCAGAAAGCTCGAGCTTTCTGGCGACATGTAGGACTTTTTG |
| Sh-MEK1#1 R | AATTCAAAAAGTCCTACATGTCGCCAGAAAGCTCGAGCTTTCTGGCGACATGTAGGAC |
| Sh-MEK1#3 F | CCGGAGTTAGCATTGCTGTAATAAACTCGAGTTTATTACAGCAATGCTAACTTTTTTG |
| Sh-MEK1#3 R | AATTCAAAAAAGTTAGCATTGCTGTAATAAACTCGAGTTTATTACAGCAATGCTAACT |
| Sh-MEK2#1 F | CCGGTTTGAACTCCTGGACTATATTCTCGAGAATATAGTCCAGGAGTTCAAATTTTTG |
| Sh-MEK2#1 R | AATTCAAAAATTTGAACTCCTGGACTATATTCTCGAGAATATAGTCCAGGAGTTCAAA |
| Sh-MEK2#2 F | CCGGTTCCAGGAGTTTGTCAATAAACTCGAGTTTATTGACAAACTCCTGGAATTTTTG |
| Sh-MEK2#2 R | AATTCAAAAATTCCAGGAGTTTGTCAATAAACTCGAGTTTATTGACAAACTCCTGGAA |
| sg-CDK4#1 F | CACCGTACCTCTCGATATGAGCCAG |
| sg-CDK4#1 R | AAACCTGGCTCATATCGAGAGGTAC |
| sg-CDK4#2 F | CACCGAAATTGGTGTCGGTGCCTAT |
| sg-CDK4#2 R | AAACATAGGCACCGACACCAATTTC |
