# Supplementary figures and images for: Domperidone inhibits cell proliferation via targeting MEK and CDK4 in esophageal squamous cell carcinoma
Source: Cancer Cell Int. 2024 Mar 25;24:114. doi: 10.1186/s12935-024-03291-8 (PMC10964662; doi:10.1186/s12935-024-03291-8)

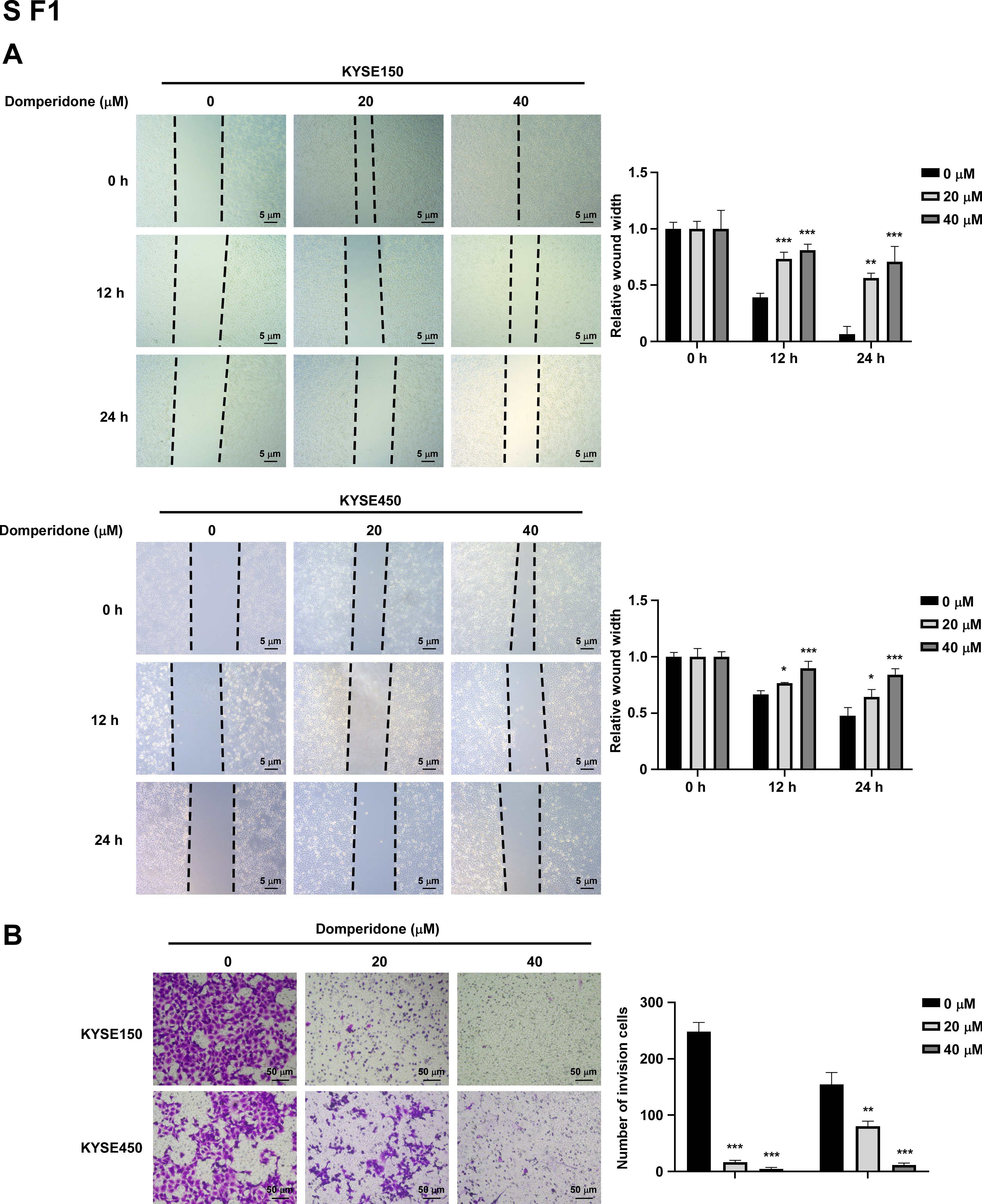

Supplement: Supplementary file 2 — Supplementary Material 2 [file 12935_2024_3291_MOESM2_ESM.png]

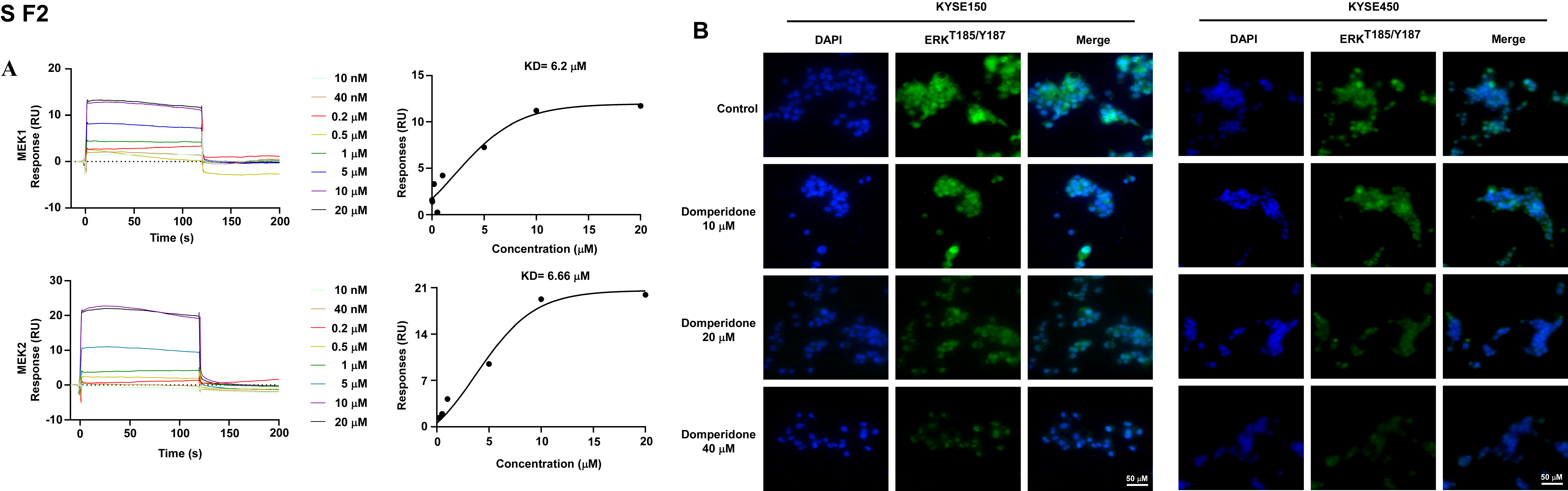

Supplement: Supplementary file 3 — Supplementary Material 3 [file 12935_2024_3291_MOESM3_ESM.png]

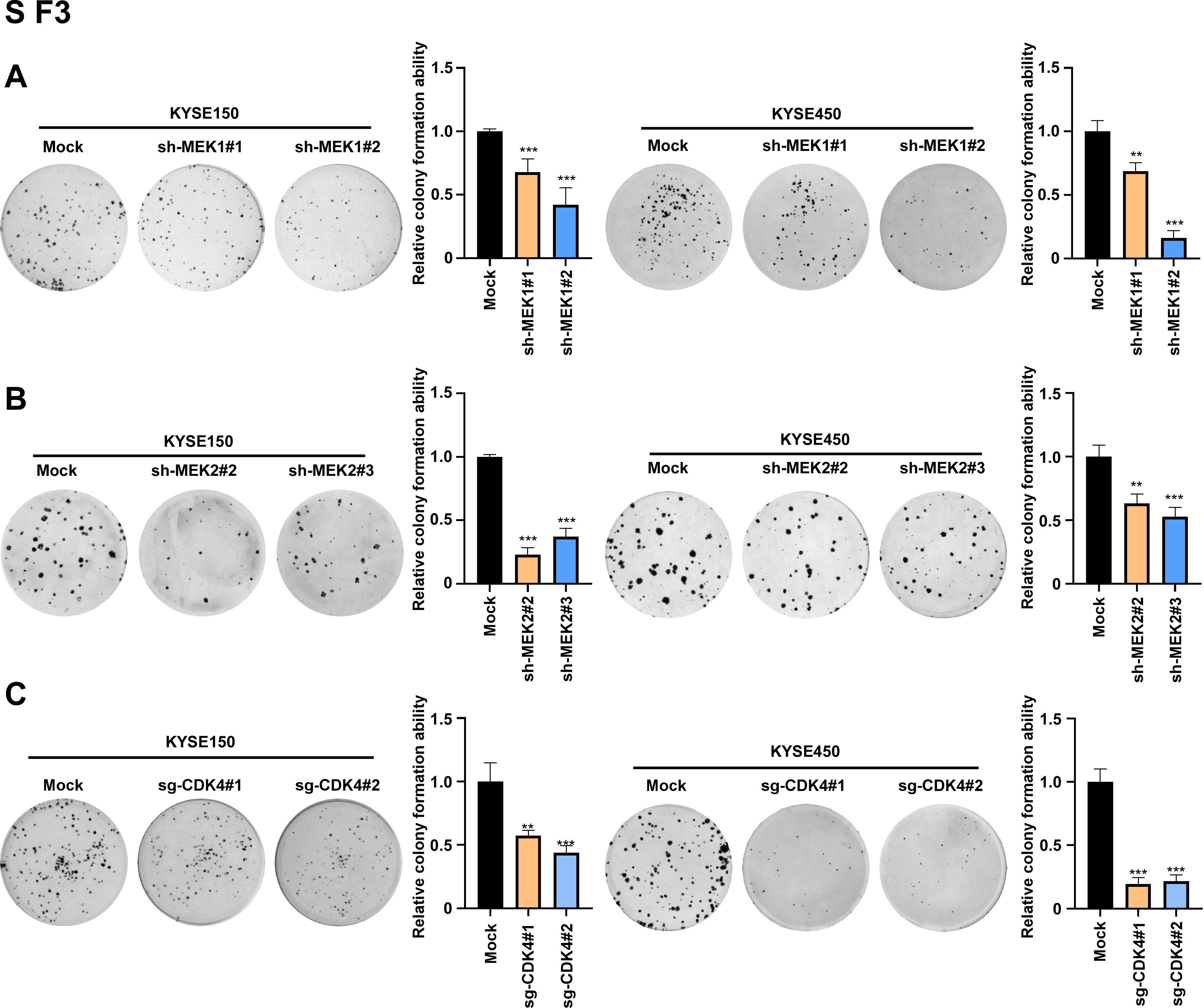

Supplement: Supplementary file 4 — Supplementary Material 4 [file 12935_2024_3291_MOESM4_ESM.png]

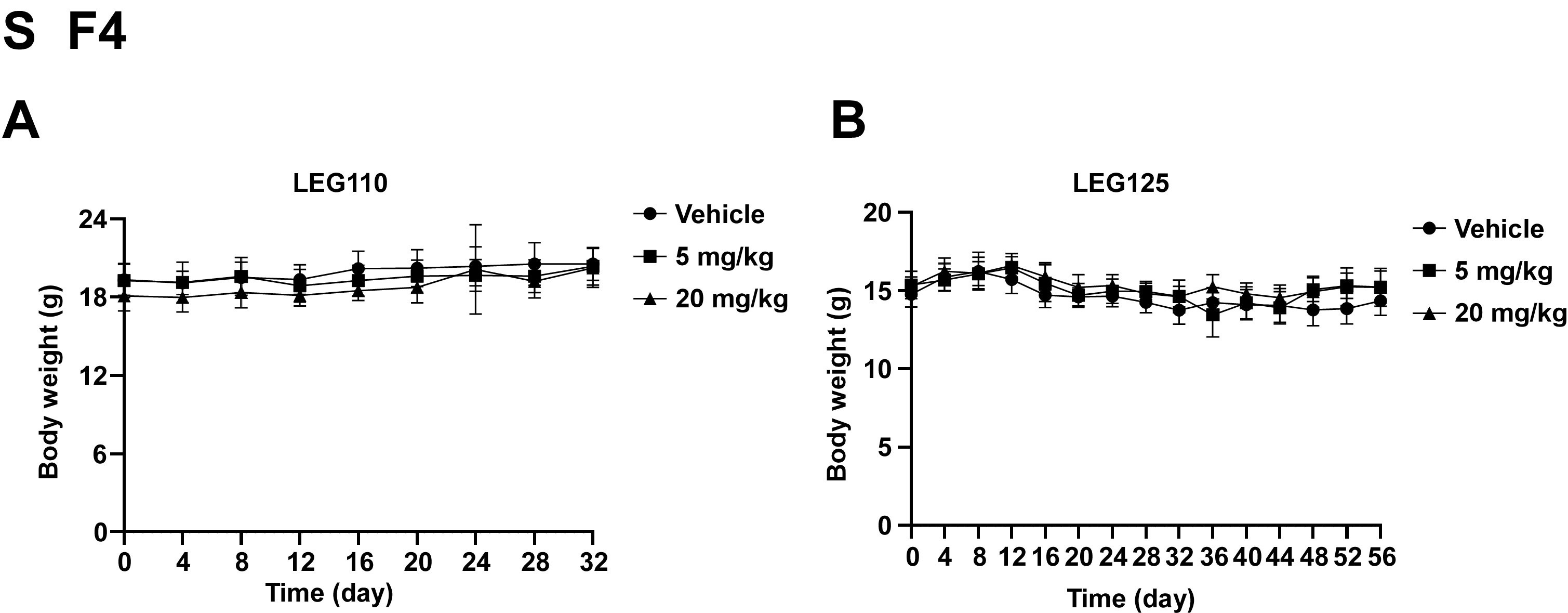

Supplement: Supplementary file 5 — Supplementary Material 5 [file 12935_2024_3291_MOESM5_ESM.png]

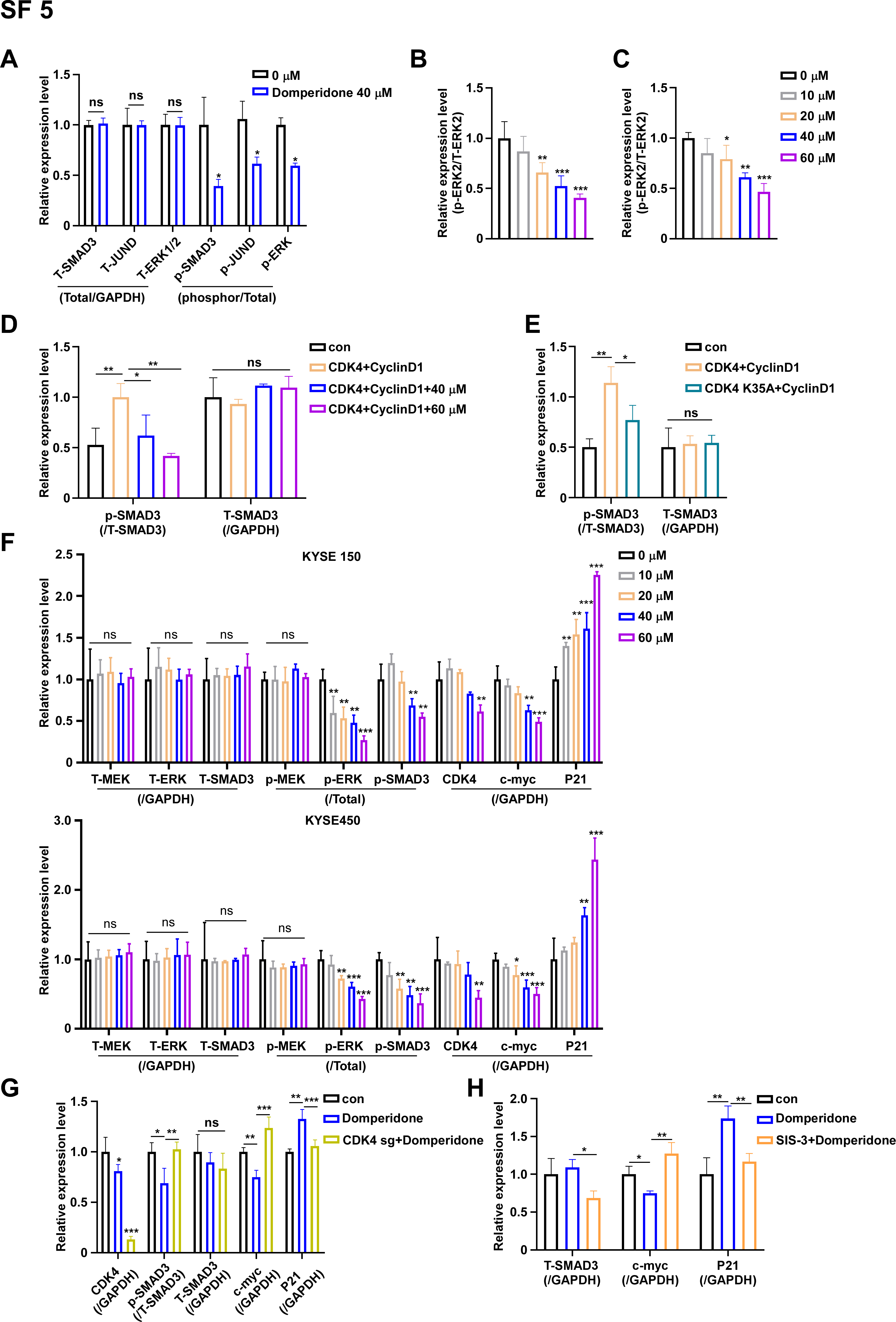

Supplement: Supplementary file 6 — Supplementary Material 6 [file 12935_2024_3291_MOESM6_ESM.png]
